# Supplementary material for: Second-Trimester Constituents of the Metabolic Syndrome and Pregnancy Outcome: An Observational Cohort Study
Source: Nutrients. 2022 Jul 18;14(14):2933. doi: 10.3390/nu14142933 (PMC9325303; doi:10.3390/nu14142933)
Supplement: Supplementary file 1 [file nutrients-14-02933-s001.zip › Supplemental Table S2.pdf]

			GDM		OR	*aOR*
**I. PE (n= 97 (4.8%))**			**-**	**+**		
		**Total**	67/1465 (4.6%)	30/576 (5.2%)	1.15 (CI 0.74-1.78)	*1.18 (CI 0.70-1.97)*
**Hypertension**	-	50/1682 (3.0%)	33/1216 (2.7%)	17/466 (3.6%)	1.36 (CI 0.75-2.46)	*1.27 (CI 0.67-2.41)*
	+	47/358 (13.1%)	34/249 (13.7%)	13/109 (11.9%)	0.86 (CI 0.43-1.70)	*1.20 (CI 0.49-2.93)*
OR		**4.93 (CI 3.25-7.48)**	**5.67 (CI 3.44-9.35)**	**3.58 (CI 1.68-7.61)**		
aOR		***3.14 (CI 1.94-5.10)***	***3.61 (CI 2.04-6.40)***	*2.02 (CI 0.79-5.20)*		
**Obesity**	-	69/1416 (4.9%)	54/1103 (4.9%)	15/313 (4.8%)	0.98 (CI 0.54-1.76)	*0.96 (CI 0.49-1.89)*
	+	28/618 (4.5%)	13/358 (3.6%)	15/260 (5.8%)	1.63 (CI 0.76-3.48)	*1.92 (CI 0.80-4.61)*
OR		0.93 (CI 0.59-1.45)	0.73 (CI 0.40-1.36)	1.22 (CI 0.58-2.54)		
aOR		*0.79 (0.47-1.31)*	*0.60 (CI 0.28-1.18)*	*1.43 (CI 0.58-3.52)*		
**Dyslipidemia**	-	84/1923 (4.4%)	59/1397 (4.2%)	25/526 (4.8%)	1.13 (CI 0.70-1.83)	*1.29 (CI 0.75-2.22)*
	+	12/117 (10.3%)	7/67 (10.4%)	5/50 (10.0%)	0.95 (CI 0.28-3.20)	*0.64 (CI 0.13-3.21)*
OR		**2.50 (CI 1.33-4.73)**	**2.65 (CI 1.16-6.04)**	2.23 (CI 0.81-6.10)		
aOR		***2.25 (CI 1.09-4.73)***	***3.07 (CI 1.26-7.50)***	*1.05 (CI 0.25-4.50)*		
**Proteinuria**	-	86/1968 (4.4%)	62/1419 (4.4%)	24/549 (4.4%)	1.00 (CI 0.62-1.62)	*1.04 (CI 0.60-1.79)*
	+	10/26 (38.5%)	4/15 (26.7%)	6/11 (54.5%)	3.30 (CI 0.64-17.16)	*11.69 (CI 0.61-225.96)*
OR		**13.68 (CI 6.03-31.03)**	**7.96 (CI 2.46-25.70)**	**26.25 (CI 7.48-92.11)**		
aOR		***12.64 (CI 4.37-36.54)***	***4.33 (CI 0.96-19.63)***	***56.85(CI9.02-358.46)***		
**≥ 2 constituents**	-	73/1794 (4.1%)	54/1326 (4.1%)	19/468 (4.1%)	1.00 (CI 0.59-1.70)	-
	+	22/191 (11.5%)	11/103 (10.7%)	11/88 (12.5%)	1.20 (CI 0.49-2.91)	-
**OR**		**3.07 (CI 1.86-5.07)**	**2.82 (CI 1.42-5.57)**	**3.38 (CI 1.55-7.37)**		
**II. <37 (n=169 (8.3%))**		**Total**	113/1465 (7.7%)	56/576 (9.7%)	1.29 (CI 0.92-1.80)	*1.45 (CI 0.99-2.12)*
**Hypertension**	-	115/1682 (6.8%)	77/1216 (6.3%)	38/466 (8.2%)	1.31 (CI 0.88-1.97)	*1.35 (CI 0.87-2.12)*
	+	54/358 (15.1%)	36/249 (14.5%)	18/109 (16.5%)	1.17 (CI 0.63-2.17)	*1.91 (CI 0.90-4.08)*
OR		**2.42 (CI 1.71-3.42)**	**2.50 (CI 1.64-3.81)**	**2.23 (CI 1.22-4.08)**		
aOR		***2.17 (CI 1.46-3.23)***	***2.13 (CI 1.31-3.47)***	***2.23 (CI 1.12-4.48)***		
**Obesity**	-	118/1416 (8.3%)	88/1103 (8.0%)	30/313 (9.6%)	1.22 (CI 0.79-1.89)	*1.66 (CI 1.03-2.67)*
	+	50/618 (8.1%)	24/358 (6.7%)	26/260 (10.0%)	1.55 (CI 0.87-2.76)	*1.27 (CI 0.68-2.38)*
OR		0.97 (CI 0.69-1.37)	0.83 (CI 0.52-1.32)	1.05 (CI 0.60-1.82)		
aOR		*0.99 (CI 0.68-1.44)*	*0.97 (CI 0.59-1.59)*	*0.81 (CI 0.42-1.54)*		
**Dyslipidemia**	-	152/1923 (7.9%)	105/1397 (7.5%)	47/526 (8.9%)	1.21 (CI 0.84-1.73)	*1.44 (CI 0.96-2.14)*
	+	17/117 (14.5%)	8/67 (11.9%)	9/50 (18.0%)	1.62 (CI 0.58-4.55)	*1.94 (CI 0.49-7.58)*
OR		**1.98 (CI 1.15-3.40)**	1.67 (CI 0.78-3.59)	**2.24 (CI 1.02-4.89)**		
aOR		***1.69 (0.92-3.11)***	*1.61 (CI 0.70-3.67)*	*1.61 (CI 0.62-4.20)*		
**Proteinuria**	-	154/1968 (7.8%)	104/1419 (7.3%)	50/549 (9.1%)	1.27 (CI 0.89-1.80)	*1.39 (CI 0.94-2.04)*
	+	10/26 (38.5%)	5/15 (33.3%)	5/11 (45.5%)	1.67 (CI 0.34-8.26)	*5.11 (CI 0.24-108.70)*
OR		**7.36 (CI 3.29-16.50)**	**6.32 (CI 2.12-18.84)**	**8.32 (CI 2.45-28.22)**		
aOR		***4.72 (CI 1.67-13.35)***	*2.10 (CI 0.43-10.20)*	***14.41 (CI 2.57-80.78)***		
**≥ 2 constituents**	-	136/1794 (7.6%)	96/1326 (7.2%)	40/468 (8.5%)	1.20 (CI 0.82-1.76)	-
	+	27/191 (19.4%)	12/103 (11.7%)	15/88 (17.0%)	1.56 (CI 0.69-3.54)	-
OR		**2.01 (CI 1.29-3.13)**	1.69 (CI 0.89-3.19)	**2.20 (CI 1.16-4.18)**		
**III. <34 (n=55 (2.7%))**			**GDM**		**OR**	***aOR***
			**-**	**+**		
		**Total**	39/1465 (2.7%)	16/576 (2.8%)	1.05 (CI 0.58-1.89)	*1.23 (CI 0.64-2.34)*

**Hypertension**	-	33/1682 (2.0%)	25/1216 (2.1%)	8/466 (1.7%)	0.83 (CI 0.37-1.86)	*0.99 (CI 0.43-2.29)*
	+	22/358 (6.1%)	14/249 (5.6%)	8/109 (7.3%)	1.33 (CI 0.54-3.27)	*1.74 (CI 0.56-5.47)*
OR		**3.27 (CI 1.88-5.68)**	**2.84 (CI 1.45-5.54)**	**4.54 (CI 1.66-12.37)**		
aOR		***2.96 (CI 1.59-5.53)***	***2.50 (CI 1.14-5.46)***	***4.05 (CI 1.35-12.19)***		
**Obesity**	-	39/1416 (2.8%)	31/1103 (2.8%)	8/313 (2.6%)	0.91 (CI 0.41-1.99)	*1.22 (CI 0.53-2.83)*
	+	16/618 (2.6%)	8/358 (2.2%)	8/260 (3.1%)	1.39 (CI 0.51-3.75)	*1.30 (CI 0.46-3.67)*
OR		0.94 (CI 0.52-1.69)	0.79 (CI 0.36-1.74)	1.21 (CI 0.45-3.27)		
aOR		*1.02 (CI 0.55-1.91)*	*0.98 (CI 0.43-2.24)*	*0.85 (CI 0.28-2.55)*		
**Dyslipidemia**	-	49/1923 (2.5%)	36/1397 (2.6%)	13/526 (2.5%)	0.96 (CI 0.50-1.82)	*1.21 (CI 0.61-2.40)*
	+	6/117 (5.1%)	3/67 (4.5%)	3/50 (6.0%)	1.36 (CI 0.26-7.05)	*1.36 (CI 0.09-20.47)*
OR		2.07 (CI 0.87-4.93)	1.77 (CI 0.53-5.91)	2.52 (CI 0.69-9.15)		
aOR		*1.90 (CI 0.76-4.76)*	*1.86 (0.54-6.42)*	*1.71 (CI 0.39-7.43)*		
**Proteinuria**	-	48/1968 (2.4%)	33/1419 (2.3%)	15/549 (2.7%)	1.18 (CI 0.64-2.19)	*1.24 (CI 0.64-2.40)*
	+	5/26 (19.2%)	4/15 (26.7%)	1/11 (9.1%)	0.28 (CI 0.03-2.89)	*NA*
OR		**9.52 (CI 3.45-26.32)**	**15.27 (CI 4.62-50.47)**	3.56 (CI 0.43-29.62)		
aOR		***5.16 (CI 1.33-20.08)***	***6.12 (CI 1.19-31.38)***	*3.62 (CI 0.29-45.45)*		
**≥ 2 constituents**	-	40/1794 (2.2%)	30/1326 (2.3%)	10/468 (2.1%)	0.94 (CI 0.46-1.95)	-
	+	13/191 (6.8%)	7/103 (6.8%)	6/88 (6.8%)	1.00 (CI 0.32-3.11)	-
OR		**3.20 (CI 1.68-6.10)**	**3.15 (CI 1.35-7.36)**	**3.35 (CI 1.19-9.47)**		

Table S2. *Odds ratios for the development of PE and delivery <37 weeks and <34 weeks. In vertical direction, ORs are calculated to explore the effect of the constituents for the group as a whole and sub-grouped without and with GDM. Along the horizontal axis, ORs are also detailed to explore an effect of GDM. Adjustments were made for the other constituents of the metabolic syndrome, age, Northern European ethnicity and gestational age at time of OGTT.*
