# Supplementary figures and images for: Second-Trimester Constituents of the Metabolic Syndrome and Pregnancy Outcome: An Observational Cohort Study
Source: Nutrients. 2022 Jul 18;14(14):2933. doi: 10.3390/nu14142933 (PMC9325303; doi:10.3390/nu14142933)

Supplemental Figure S1

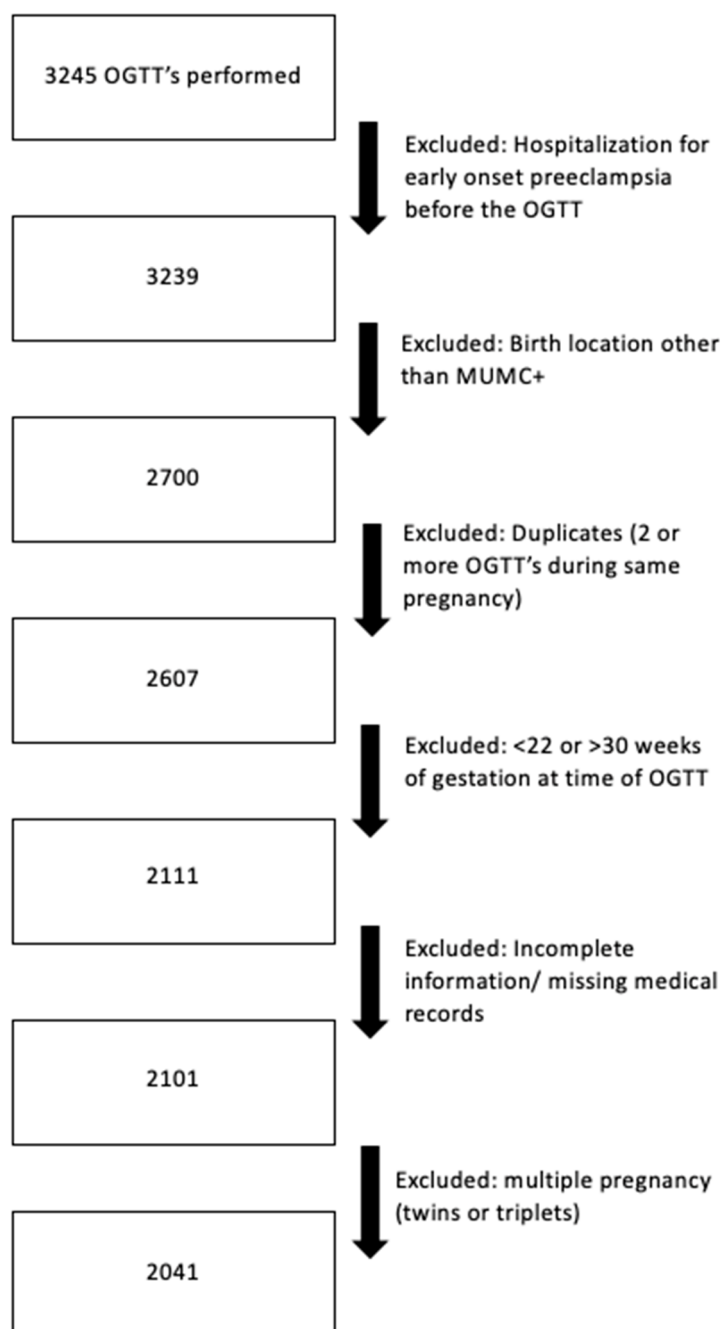

Supplemental Figure S1. *Exclusion procedure*

Supplement: Supplementary file 1 [file nutrients-14-02933-s001.zip › Supplemental Figure S1.pdf]
